# Effect of Low- and High-Si/Al Synthetic Zeolites on the Performance of Renovation Plasters

**DOI:** 10.3390/ma18204710

**Published:** 2025-10-14

**Authors:** Joanna Styczeń, Jacek Majewski

**Affiliations:** 1 Faculty of Civil Engineering and Architecture, Lublin University of Technology, 40 Nadbystrzycka Str., 20-618 Lublin, Poland; 2 Faculty of Electrical Engineering and Computer Science, Lublin University of Technology, 38A Nadbystrzycka Str., 20-618 Lublin, Poland; j.majewski@pollub.pl

**Keywords:** zeolite, synthetic zeolites, cement matrix microstructure, renovation plaster, moisture passive regulation, water vapour permeability, electrical resistivity

## Abstract

The appropriate selection of renovation plaster properties is essential for ensuring the durability and effectiveness of conservation works. This study focused on the design and characterization of cement-based renovation mortars modified with synthetic zeolites with different Si/Al ratios. It was assumed that high-silica zeolites would provide more favorable mechanical and hygric performance than low-silica types. Owing to their porous structure and pozzolanic reactivity, zeolites proved to be effective additives, enhancing both the microstructure and functionality of the mortars. The modified mixtures exhibited increased total porosity, higher capillary absorption, and improved moisture transport compared with the reference mortar based on CEM I 52.5R. Dynamic vapor sorption tests confirmed that the zeolite-containing mortars achieved Moisture Buffer Values (MBV) above 2.0 g/m^2^, which corresponds to the “excellent” moisture buffering class. Electrical resistivity measurements further demonstrated the relationship between denser microstructure and enhanced durability. At the frequency of 10 kHz, the electrical resistivity of the reference mortar reached 43,858 Ω·m, while mortars with 15% ZSM-5 and 15% Na-A achieved 62,110 Ω·m and 21,737 Ω·m. These results show that the addition of high-silica zeolite promotes the formation of a denser and more insulating matrix, highlighting the potential of this method for non-destructive quality assessment. The best overall performance was observed in mortars containing the high-silica zeolite ZSM-5. A 35% replacement of cement with ZSM-5 increased compressive strength by 10.5% compared with the reference mortar R (4.3 MPa). Frost resistance tests showed minimal mass loss (0.03% at 15% and 1.79% at 35% replacement), and ZSM-5 mortars also maintained integrity under salt crystallization. These improvements were attributed to the reaction of reactive SiO_2_ and Al_2_O_3_ from the zeolites with Ca(OH)_2_, leading to the formation of additional C-S-H. A higher Si/Al ratio promoted a denser, fibrous C-S-H morphology, as confirmed by SEM, which explains the improved strength and durability of mortars modified with ZSM-5.

## 1. Introduction

Historic buildings often struggle with problems of moisture ingress and salt contamination in masonry, which leads to the degradation of plasters and paint coatings. Simple wall drying combined with the application of conventional lime–cement plasters proves insufficient due to their low resistance to crystallizing salts [1]. Such plasters degrade within a few months (cracking, detachment from the substrate, discoloration). In such cases, the most effective solution is the application of horizontal and vertical damp-proof insulation, wall drying, and the use of specialized renovation plaster. Renovation plasters must fulfill a set of material requirements defined by WTA guidelines and standards. Above all, it should be characterized by high porosity (a macroporous structure) to provide sufficient space for salt crystallization and ice formation in the pores, which ensures resistance to salt crystallization pressure and frost durability of the mortar. Limited capillary water uptake is equally important, since sufficiently low capillary absorption reduces the transport of harmful salts from the substrate into the outer plaster layer [2,3]. To meet such strict requirements, renovation mortars are increasingly modified with additives designed to alter their microstructure, such as lightweight aggregates and porous materials with sorptive properties towards water. One promising solution is the incorporation of zeolites as functional additives to mineral mortars [4].

Zeolites are crystalline aluminosilicates with a porous structure and a well-developed network of two- and three-dimensional channels [5]. They are characterized by high sorption capacity and significant pozzolanic reactivity owing to the presence of reactive silica and alumina [6]. Previous studies have confirmed that zeolites synthesized from fly ash exhibit tailored textural properties and chemical composition, which make them suitable as functional components in construction materials. The addition of zeolites to cementitious materials can beneficially influence their performance, including strength, durability, frost resistance, and water absorption. Zeolite proved to be a more effective additive in renovation renders than commonly used perlite [7].

To date, studies on renovation plasters incorporating zeolites remain limited. Barnat-Hunek et al. [8] designed plasters containing natural zeolite (clinoptilolite) combined with lightweight aggregate (expanded clay). All tested mortars exhibited high porosity and significant capillary absorption. The strongest improvement of these parameters was observed for the zeolite-modified mixes, which effectively absorbed water and accumulated crystallizing salts within their pores. All compositions also showed high frost resistance. Sezemanas et al. [9] investigated the effect of natural clinoptilolite (15% sand replacement) on plaster properties applied on autoclaved aerated concrete. The addition of zeolite increased density and refined the plaster microstructure, which translated into improved compressive and flexural strength as well as better adhesion to the aerated concrete substrate [10].

The moisture-related properties of renovation plasters play a crucial role in their performance during service. These properties can be divided into static (e.g., water vapor permeability coefficient, sorption isotherm) and dynamic (moisture buffering capacity, adsorption–desorption cycles). In particular, dynamic parameters are currently the subject of intensive research, as they are used to assess the ability of porous materials to passively regulate indoor air humidity. The incorporation of materials with high hygroscopicity and large moisture-buffering capacity may mitigate fluctuations of relative humidity, thereby reducing the load on air circulation systems and limiting the risk of condensation and mold growth. Ranesi et al. [11] examined different traditional plasters (clay, lime, gypsum) regarding their suitability for humidity regulation. They demonstrated that, although clay-based materials exhibit favorable sorption properties, typical cement plasters attain significantly lower sorption values and cannot be classified into the highest moisture buffering classes (MBV). The Moisture Buffer Value (MBV) describes the capacity of a material to buffer humidity, and its effectiveness in passively regulating daily fluctuations of indoor relative humidity. In simple terms, MBV corresponds to the amount of water vapor adsorbed (or released) per 1 m^2^ of exposed surface during cyclic daily humidity variations from high to low. McGregor et al. [12] showed that unfired clay can achieve a significantly higher MBV than traditional building materials, which has a positive effect on indoor microclimate and increases the durability of building materials. Another simulation-based study reported that the use of materials with high MBV can reduce energy consumption for air-conditioning by up to 25–30% in buildings located in temperate and semi-arid climates [13].

In recent years, there has also been growing interest in the electrical parameters of building materials, such as electrical resistivity, conductivity, and dielectric permittivity. Electrical resistivity is widely used to assess the effect of additives and modifications ranging from nanosilica, fly ash, LC3, recycled components, and coatings to conductive admixtures. An increase in resistivity is often interpreted as a “densification” of the microstructure and improved resistance to carbonation and chloride ingress, while a decrease can indicate enhanced conductivity beneficial for heating applications or self-sensing materials [14,15]. Electrical resistivity measurements are non-destructive, simple, and rapid, while simultaneously providing information linked to the microstructure and material properties (e.g., porosity, degree of cement hydration, ionic concentration in pore solution). In the case of cementitious composites, resistivity has been shown to correlate with durability and strength—as permeability (porosity) decreases, both electrical resistivity and compressive strength increase [16]. For this reason, electrical resistivity measurements were also included in this study as a complementary tool for assessing the microstructure and performance of the investigated plasters.

Up to now, research on renovation plasters has predominantly focused on physical–mechanical properties, such as density, compressive and flexural strength, water absorption, frost resistance, and adhesion to the substrate. Much less attention has been paid to moisture-related (sorption) parameters and electrical properties. Comprehensive studies comparing these characteristics, especially for mortars modified with unconventional additives, are lacking. Considering the porous structure of zeolites, it can be expected that such additives will enhance the sorption capacity of renovation mortars, potentially leading to more effective drying of damp masonry and passive regulation of indoor air humidity. Therefore, it is justified to investigate both moisture-related and electrical properties of zeolite-modified plasters. In the available literature, research has mainly concerned natural zeolite additives, whose properties are predetermined by geological origin. A novel approach may involve the use of synthetic zeolites derived from fly ash, since their structure and chemical composition can be tailored already at the synthesis stage to achieve desired features, such as controlled porosity in the final renovation plaster. It is also important to note that zeolite minerals can be successfully synthesized from fly ash by-product of coal combustion. Fly ash is rich in SiO_2_ and Al_2_O_3_, which makes it an excellent raw material for zeolite synthesis [17]. This represents a solution that is both economically and environmentally beneficial (secondary utilization of waste material) [18,19].

In this study, the influence of the Si/Al ratio in the zeolite framework on the technological performance of renovation plasters was examined, with particular emphasis on their electrical properties. Although zeolites have already been studied as mineral admixtures in mortars and concretes, the specific role of the Si/Al ratio in determining their performance remains poorly addressed. Previous research mainly focused on natural clinoptilolite, while systematic comparisons between low- and high-Si/Al synthetic zeolites are lacking. Investigating this parameter is essential, since it directly affects the pozzolanic reactivity, ion-exchange capacity, and microstructural densification of cementitious systems. Therefore, this work provides a novel contribution by evaluating how zeolites with contrasting Si/Al ratios, Na-A and ZSM-5, impact the mechanical, hygric, and electrical behavior of renovation plasters.

## 2. Materials and Methods

### 2.1. Materials

Seven formulations of renovation plasters (storage layer) were designed for the experimental program. The reference composition of all mixtures included Portland cement CEM I 52.5R as the primary binder, hydrated lime, quartz sand with a particle size of 0–2 mm, and lightweight mineral additives in the form of expanded clay and clinoptilolite. In addition, admixtures of methylcellulose and ethylene–vinyl acetate copolymer were incorporated to improve the workability and performance properties of the mortars. In the reference plaster (denoted as P), no pozzolanic additive in the form of zeolite was introduced into the cement binder. In the other six formulations, a portion of the cement was replaced with synthetic zeolite powder. Two types of zeolites were applied: Na-A (low-silica zeolite with a low Si/Al ratio) and ZSM-5 (high-silica zeolite with a high Si/Al ratio). For each zeolite type, three replacement levels were used: 15%, 35%, and 50% (percentage by mass of cement replaced with zeolite). The replacement levels were selected based on previous studies on zeolite-modified mortars. The reduction in cement content in each mixture was compensated by an equivalent mass of zeolite. The designations of the mortars correspond to the type and dosage of zeolite used (e.g., Na-A 15% denotes a plaster with 15% Na-A zeolite addition). The compositions of all seven mortars are summarized in Table 1. For the experimental tests, a series of cuboid samples with dimensions of 40 mm × 40 mm × 160 mm was prepared in accordance with EN 1015-11:1999 [20].

**Figure 1:**
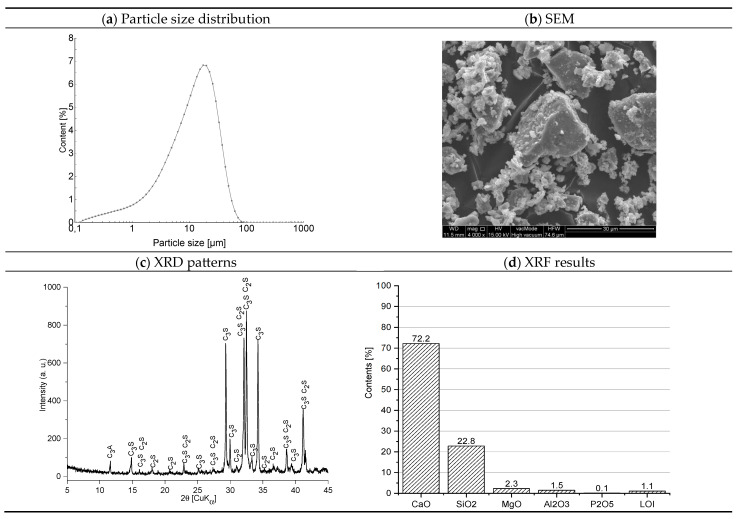
Structure and properties of cement [22].

Portland cement CEM I 52.5R (Cemex Poland, Chełm, Poland, Chełm cement plant) was used as the primary binder. The cement was tested in accordance with EN 1015-2:1998 [21]. Its main properties are as follows: initial setting time—160 min, final setting time—210 min, loss on ignition—2.77%, compressive strength after 2 days—31.6 MPa, and compressive strength after 28 days—64.7 MPa. The chemical composition and fundamental physical parameters of the cement are presented in Figure 1.

The findings regarding the granular composition of the cement show a distinctly monomodal distribution pattern (Figure 1a). The composition predominantly comprises particulates exhibiting sizes within the interval of 0.01–20.0 µm, constituting approximately 85.82% of all grains. Morphological characteristics of cement crystals (Figure 1b) show individual grains of cement phases with irregular sharp-edged shapes. Their size rarely exceeds 30 µm. The diffractogram of the mineral composition of the cement is shown in Figure 1c. The main crystalline phases of the cement were recognized by the characteristic interplane distances dhkl. The following were recognized in the mineral composition of the cement: alite (C3S)—dhkl: 2.78; 2.76; 3.05; 2.61; 2.74 Å, belite (C2S)—dhkl: 2.78; 2.79; 2.75; 2.62; 2.72 Å, and tricalcium aluminate (C3A) dhkl: 7.61 Å. The chemical composition of the cement is shown in Figure 1d. Calcium oxide (72.4%) and silicon oxide (19.4%) dominate.

Two zeolites from different structural groups, the Na-A and the ZSM-5, were selected for the study. They differ in their Si/Al ratio and three-dimensional channel system. The Si/Al ratio in the zeolite framework plays a crucial role in the hydration process. The use of zeolites with different Si/Al ratios made it possible to determine the effect of this parameter on the physico-mechanical properties of the plasters, including adsorptive and desorptive dynamic behavior, moisture buffering capacity, and electrical resistivity. The zeolites were synthesized via a two-step hydrothermal process. In the first stage, fly ash zeolite was obtained, yielding a post-reaction solution. This solution contained significant amounts of silicon and aluminum, the essential elements for the construction of zeolitic frameworks, and was subsequently used in the second stage. The synthesis conditions of the zeolites are summarized in Table 2b.

Based on the SEM analysis (Table 2a), it was observed that the Na-A zeolite crystals exhibit a regular cubic morphology with sizes ranging from 0.5 µm to 3 µm, whereas the ZSM-5 zeolite, belonging to the pentasil group, is characterized by a plate-like morphology. The grains of this material are very fine and rarely exceed 1.5 µm. Furthermore, the ZSM-5 rarely occurs as individual crystals; its particles are often intergrown, forming irregular aggregates.

For the Na-A zeolite, three particle-size fractions were identified (Table 2c): 0.01–2 µm (23.91%), 2–20 µm (32.43%), and 50–250 µm (32.92%), corresponding to maxima of 1.5, 12, and 90 µm, respectively. ZSM-5 zeolite exhibited the finest particle size distribution among the tested zeolites, dominated by two fractions, 0.01–2 µm (21.70%) and 2–20 µm (71.86%).

**Figure 2:**
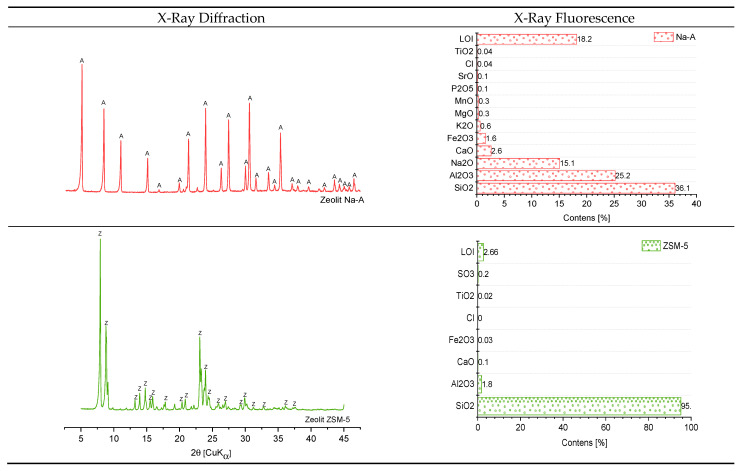
Structure and properties of zeolites. A—reflections of zeolite Na-A; Z—reflections of zeolite ZSM-5.

The mineral composition of the zeolites, determined by XRD, is shown in Figure 2. The presence of the Na-A phase was confirmed by the occurrence of characteristic reflections for this zeolite at dhkl = 12.31, 8.70, 2.98, 3.71, and 3.29 Å. The chemical composition determined by XRF (Figure 2) revealed that the Na-A zeolite is dominated by silica and alumina, accounting for 36.1% and 25.2%, respectively. In contrast, the ZSM-5 is a highly siliceous zeolite, with SiO_2_ content reaching 95.0%. Similar to the other zeolitic forms, the XRD pattern of the ZSM-5 displayed only reflections assigned to the zeolitic phase, indicating its high purity (dhkl = 11.13, 9.94, 10.05, 11.15, and 9.75 Å).

### 2.2. Methodology

Sample preparation: All mortars were prepared with a constant water-to-cement ratio of 0.5. The fresh plaster mixtures were cast into prismatic specimens with dimensions of 40 mm × 40 mm × 160 mm. The specimens were kept in molds under foil for the first 2 days to ensure moist curing conditions, and then demolded and cured for 28 days under laboratory conditions (temperature 20 ± 1 °C, relative humidity 50%).

Adsorptive and desorptive dynamic properties—to assess the dynamic moisture sorption properties of the materials, a test procedure based on DIN 18947:2024 [23] was adopted. All seven materials were tested as single cuboid samples to determine their hygroscopic behavior under a step change in ambient humidity. The adsorption phase was conducted in a climate chamber (KBK-100, Wamed, Warsaw, Poland) with internal dimensions of 550 mm × 550 mm × 360 mm (volume 110 dm^3^). Prior to testing, samples were preconditioned for one week in a controlled laboratory environment (20 ± 2 °C, 48–55% RH) to reach near-equilibrium. They were placed on an open-grid tray for free air access on all sides and weighed daily. When the day-to-day mass change fell below 10 mg (depending on sample composition), the sides and bottom of each cuboid were sealed with diffusion-resistant aluminum foil and tape, leaving only the top surface (40 mm × 40 mm) exposed. After preconditioning, the tray of samples was placed in the climate chamber to start the adsorption phase. The chamber was maintained at 20 ± 1 °C and 80 ± 1% RH for 12 h, with conditions monitored by three sensors. During this period, water vapor adsorption on the exposed sample surfaces caused a progressive mass increase. Sample mass was recorded after 0.5, 1, 3, 6, and 12 h of exposure (after DIN 18947 recommendations). Each weighing was done on a laboratory balance (±1 mg precision). For each measurement, samples were individually removed from the chamber for about 10 s to weigh, while the external ambient was maintained at 20 ± 1 °C and 50% RH to minimize environmental influence. Immediately after the 12 h adsorption phase, the samples were returned to laboratory ambient conditions (20 ± 2 °C, 48–55% RH) to begin the desorption phase. Sample masses were similarly recorded after 0.5, 1, 3, 6, and 12 h of desorption.

Moisture Buffering—The cyclic adsorption–desorption behavior of building materials is quantified by the Moisture Buffer Value (MBV), which measures a material’s capacity to moderate daily indoor humidity fluctuations. Simplistically, MBV is defined as the mass of moisture adsorbed or desorbed per 1 m^2^ of material surface when the ambient relative humidity cycles between a high and low set-point over a 24 h period. The MBV concept was introduced in 2005 by a NORDTEST project (2004–2005) [24], which established a standard test protocol for MBV determination. According to the NORDTEST protocol, the sample should have at least one exposed face of ≥100 cm^2^ area (with all other sides sealed, e.g., with aluminum foil). The exposed side should be ≥100 mm in length, and the sample thickness should exceed the daily moisture penetration depth. Before testing, samples are conditioned to equilibrium at 23 ± 5 °C and 50 ± 5% RH until the 24 h mass change is <0.1%. During the MBV test, samples are cycled at 23 °C between 75% RH for 8 h (moisture uptake) and 33% RH for 16 h (moisture release). These 24 h cycles are repeated until the change in mass over three consecutive cycles is less than 5%. The MBV is then calculated as the average moisture uptake/release (per unit area) during the last few stable cycles (using at least three cycles for accuracy). In this study, the same samples from the adsorption/desorption test were used for MBV determination following the NORDTEST protocol. Because each weighing required briefly removing the sample from the chamber (120 s), a small fan was installed inside the chamber to quickly re-establish the set humidity conditions after each removal. After completing the required cycles and calculations, MBVs were obtained for all samples.

**Figure 3:**
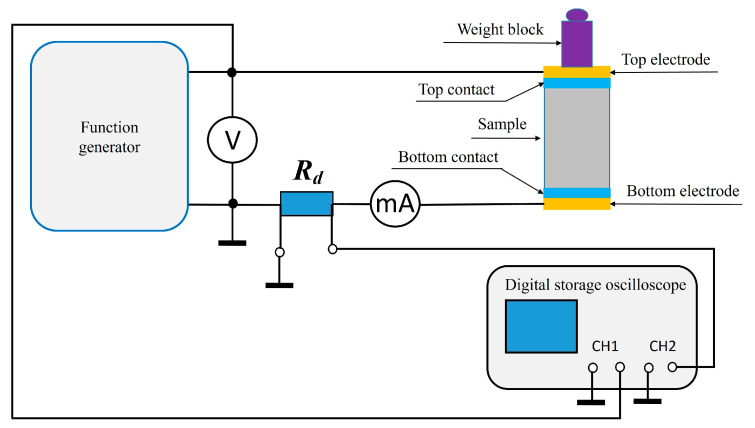
The measurement setup for determining the resistance value of a sample.

The electrical resistivity ρ was determined indirectly by measuring the electrical resistance of the specimens while accounting for their dimensions. The tests were carried out on prismatic specimens with dimensions of 40 mm × 40 mm × 160 mm. A standard two-electrode method with two plate electrodes was applied. Each specimen was placed between two electrodes made of thin, soft aluminum foil, cut to match the cross-sectional area of the specimen. To ensure proper electrical contact and minimize interfacial resistance between the electrode and the specimen, thin cotton fabric pads saturated with water were used as so-called wet contacts. A light load was placed on the top electrode to maintain constant pressure on the system. The prepared setup was then connected to an AC voltage generator with adjustable frequency. The RMS values of current and voltage were measured at different frequencies ranging from 20 Hz to 100 kHz using a digital multimeter. The resistivity of each specimen was calculated according to the formula:

*ϱ* = *R*·*A*/*L* (1)

where A is the cross-section area of the sample perpendicular to the current flow, and L is the height of the sample. The measurements were carried out at room temperature (20 ± 1 °C). Prior to electrical resistivity measurements, the mortar specimens were oven-dried at 105 °C until reaching constant mass, in order to remove free and physically bound water. This conditioning step, widely adopted in cementitious material testing [1], ensures consistent moisture content across all samples and allows reliable comparison of resistivity values. At least two independent measurements were performed for each sample. The complete measurement setup is presented in Figure 3.

The grain size distribution was determined using a Mastersizer 3000 (Malvern Instruments, Malvern, UK) based on the phenomenon of laser diffraction. The dry dispersion method was applied, and the particle sizes were analyzed within the equivalent diameter range of 0.1–1000 µm.

The X-ray diffraction (XRD) analysis was conducted to determine the phase composition and to monitor the progress of the pozzolanic reaction. The tests were carried out on powdered, cured pastes to identify changes in the hydration products up to 90 days of curing. A Panalytical X’PertPRO MPD, Westborough, MA, USA diffractometer equipped with a PW 3020 goniometer, a Cu lamp (CuKα = 1.54178 Å), and a graphite monochromator was used. Prior to testing, the pastes were ground in an agate mortar and sieved through a 63 µm mesh sieve. The prepared material was placed in aluminum holders and mounted in the diffractometer. The measurements were performed over an angular range of 5–65° 2θ, with a step size of 0.02° 2θ and a counting time of 5 s per step. The obtained diffraction data were processed using the X’Pert HighScore v 3.0 software, while the PDF-2 Release 2010 JCPDS-ICDD database was used for phase identification.

The chemical composition of the pastes was determined using X-ray fluorescence (XRF). An Epsilon 3 spectrometer (Panalytical, Malvern, UK) equipped with a Rh 9 W, 50 kV, 1 mA X-ray tube, a 4096-channel spectrum analyzer, six measurement filters (Cu-300, Cu-500, Al-50, Al-200, Ti, Ag), and a high-resolution solid-state SDD detector cooled with a Peltier cell was employed. Prior to testing, the samples were oven-dried to constant mass, ground in an agate mortar, and placed in plastic cups in amounts of approximately 5–8 g.

The microstructure of the pastes was examined using a Quanta 250 FEG, Ann Arbor, MI, USA scanning electron microscope (FEI). The instrument was equipped with a LaB_6_ cathode electron gun and an EDS system (EDAX) for elemental composition analysis. The study was carried out in high-vacuum mode, using secondary electron (SE) imaging at an accelerating voltage in the range of 10–15 kV.

The consistency of the fresh mortar and the bulk density of the fresh mortar were determined in accordance with PN-EN 1015-10:1999. The air content in fresh mortar was measured following the procedure described in PN-EN 1015-7, using the pressure method [25].

The water absorption coefficient due to capillary action of the hardened mortar was determined according to EN 1015-18:2002 [26].

The flexural strength was measured using the three-point bending test in compliance with EN 1015-11:1999 [24]. The compressive strength was determined according to EN 1015-18:2002, using the halves of 40 mm × 40 mm × 160 mm prisms remaining after the flexural strength test. For each mortar series, the reported results represent the arithmetic mean of six measurements. The tests were carried out using a CONTROLS, Tucker, GA, USA, Advantest 9 testing machine equipped with a loading frame of up to 250 kN [20].

Frost resistance was evaluated according to PN-85-B-04500:1985; Building Mortars-Testing of Physical and Mechanical Properties. PKN: Warsaw, Poland, 1985, using the standard method. The test was performed on 40 mm × 40 mm × 160 mm specimens after 28 days of curing [27].

Salt crystallization resistance was determined in accordance with EN 12370:1999, using eight cubic specimens with dimensions of 40 mm × 40 mm × 40 mm [28].

The adhesion of the plasters to the substrate was assessed according to EN 1015-12:2016 [29].

## 3. Results and Discussion

### 3.1. Physical Properties of the Renovation Plasters—Strength Features

A series of tests related to the physical properties of the renovation plasters was carried out, and the results are summarized in Table 3. All designed mortars achieved the flow range required by the WTA guidelines (170 ± 5 mm), ensuring good workability of the plaster. The reference mortar (without zeolite) reached the highest flow value (175 mm). The zeolite additions, as materials with strong water absorption capacity, caused a slight reduction in flow—with increasing zeolite content, the consistency of the mixture became denser. For instance, mortars with 50% zeolite replacement exhibited a flow value of approximately 165 mm. Nevertheless, the differences remained within the permissible range, and no difficulties were observed in the application of the fresh plasters. The air content in the fresh mortars ranged from 25% to 28% by volume (with the highest value recorded for the reference mortar), indicating a highly aerated character of all mixtures—a property desirable for renovation plasters [30].

The incorporation of synthetic zeolites had a pronounced effect on the density and porosity of the hardened plasters [31]. The reference mortar exhibited an apparent density of 1307 kg/m^3^. A 15% zeolite replacement resulted in a slight increase in density (Na-A 15%: 1332 kg/m^3^; ZSM-5 15%: 1321 kg/m^3^), which may be attributed to partial pore refinement at a low pozzolanic dosage. However, at higher replacement levels, the effect was reversed—the mortars with 35% and 50% zeolite were lighter than the reference. The lowest apparent density was recorded for plasters with 50% zeolite (1280 kg/m^3^ for both Na-A and ZSM-5).

The total porosity of the mortars ranged from approximately 44% to 52% (ZSM-5 50%). In general, higher zeolite replacement levels led to increased overall porosity [10]. he plasters with 50% ZSM-5 exhibited the highest porosity among all compositions (>50%). This increased porosity indicates the development of a larger number of capillary pores in the microstructure, which has significant implications for moisture transport.

Capillary water absorption. Increased porosity and a more developed capillary network resulted in higher water absorption capacity. The water absorption coefficient (after 24 h) for the reference mortar was 7.4 kg/m^2^, whereas for zeolite-modified mortars, it was higher and increased with zeolite content. For instance, Na-A 50% absorbed approximately 13.4 kg/m^2^, and ZSM-5 50% absorbed 14.4 kg/m^2^, representing nearly a twofold increase compared to the reference. A similar trend was observed for the capillary rise: the water front in the reference plaster reached 31 mm, while in ZSM-5 50% it extended up to 70 mm.

**Figure 4:**
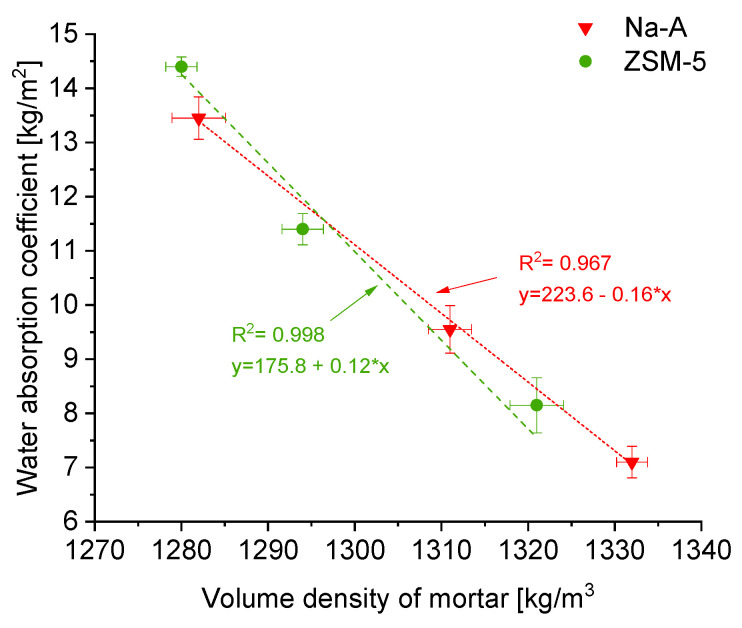
Correlation between the bulk density of hardened mortar [kg/m^3^] and the water absorption coefficient [kg/m^2^].

The relationship between density and capillary water absorption is presented in Figure 4. With decreasing apparent density (i.e., increasing porosity), the height of capillary rise increased, showing a clear linear trend. It can be concluded that zeolite incorporation, by producing a more porous structure, facilitates water transport in the capillary rise zone. From a practical standpoint, the higher capillary absorption of zeolite-modified plasters may be advantageous, as it promotes the removal of moisture from damp masonry into the plaster layer and favors the accumulation of salts within the pores of the plaster rather than in the masonry itself. In renovation plaster systems, the primary role of salt storage is performed by the base coat (porous buffer layer), which was the subject of this study.

Zeolites increase the volume of plaster mortars and their water absorption, which is associated with the higher proportion of capillary pores responsible for fluid transport. Of particular importance are the pores with capillary-active diameters, as they determine the mechanism of water migration [32]. Pores larger than 60 µm are not capillary-active. Giosuè et al. [33] reported that larger pores had a stronger influence on capillary absorption than micropores, since water initially fills the larger capillaries and subsequently the finer ones, which was also confirmed by Benachour et al. [34]. The highest capillary absorption was recorded for mortars with 50% ZSM-5 replacement. This zeolite exhibited the largest pore volume among the investigated additives (0.1 cm^3^/g), which explains the enhanced ability of the mortar to absorb and retain water. For renovation plasters, this is considered a beneficial effect, as it facilitates the migration of moisture from damp masonry and its subsequent evaporation.

**Figure 5:**
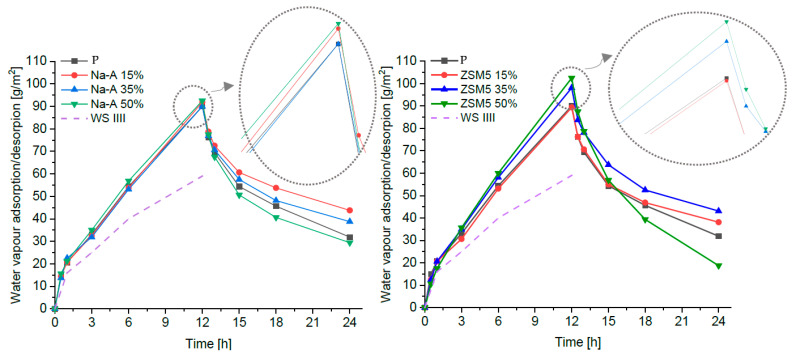
Adsorption–desorption curves of the plasters and hygroscopicity class WS III defined in DIN 18947 [23].

Figure 5 presents the adsorption and desorption curves for each plaster, along with the thresholds of the third adsorption class (WS III) defined in DIN 18947 [35]. All specimens comfortably met the DIN 18947 classification requirements for water vapor adsorption: the highest class, WS III, begins at a threshold of 60 g/m^2^ after 12 h, whereas the average mass increase in the tested set was approximately (90 ± 10) g/m^2^. The greatest sorption capacity was observed for samples with 50% zeolite replacement in cement, as both zeolitic forms significantly increased the pore volume in the plaster structure.

**Figure 6:**
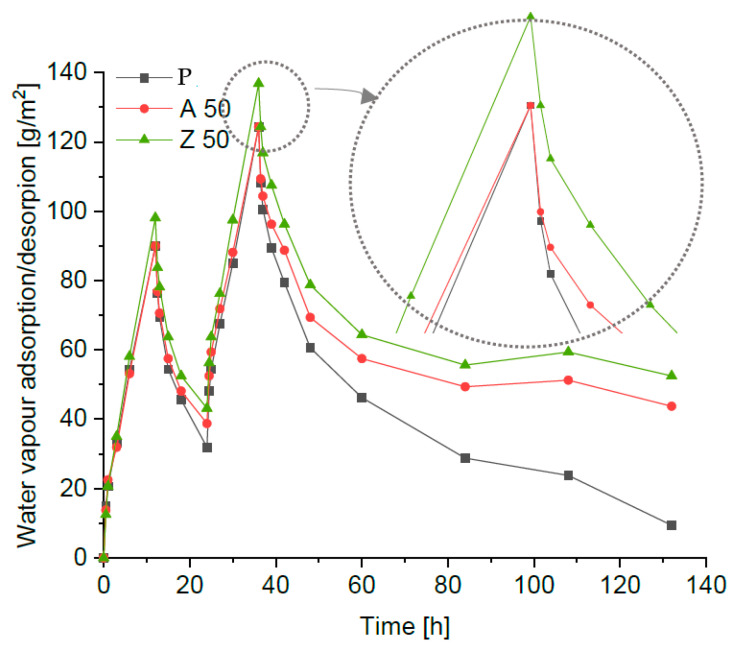
The hygroscopic behaviour of the renovation plaster.

After 12 h of the first desorption phase, the masses of all specimens were still higher than their initial values, and a second adsorption cycle followed by desorption was carried out (Figure 6). According to the NORDTEST recommendations [36], the cycles should be continued until a steady state is reached; in practice, a significant residual moisture content is often observed, which requires extending the test duration [11]. After the second desorption phase (48 h from the beginning of the test), the mass increase in all specimens was even greater than after the first cycle. Therefore, the second desorption phase was continued, with weighing performed after 24, 48, 72, and 96 h. The test procedure was terminated after 132 h. All specimens exhibited noticeable residual moisture after 96 h of the second desorption, when the curves became flat or slightly descending (for clarity, Figure 6 presents only the reference plaster and the mixtures with 50% zeolite). The reference sample showed the lowest residual moisture absorption (Figure 6), significantly lower than any of the modified samples. The plaster with 50% ZSM-5 replacement exhibited the highest hygroscopicity. A similar phenomenon—increased moisture retention during the desorption phase and pronounced sorption hysteresis related to macroporosity—was also reported by Brachaczek et al. [37] for renovation mortars and porous composites.

**Figure 7:**
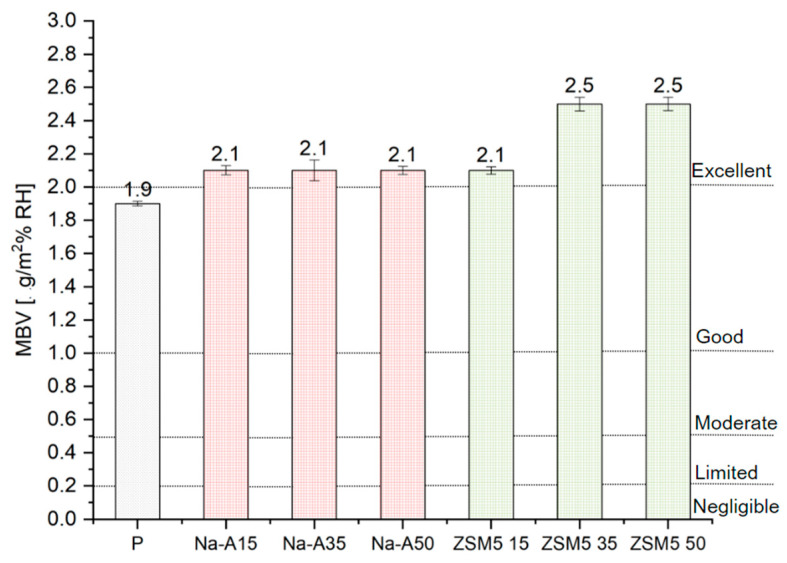
The MBVs of the tested samples (with standard deviation bars) and limits from NORDEST.

Figure 7 presents the average MBVs for adsorption and desorption calculated for all tested plasters, along with the corresponding moisture buffering classes.

The best performance was achieved by the renovation plasters with 35% and 50% ZSM-5 replacement. The addition of Na-A zeolite, regardless of dosage, increased the MBV coefficient (2.1 g/m^2^%RH) compared to the reference mortar (1.92 g/m^2^%RH). All zeolite-modified plasters fall into the ‘excellent’ class (MBV > 2 g/m^2^%RH) as proposed by Rode [38]. Ranesi et al. [11] investigated standard clay-, gypsum-, and cement-based plasters, and none of the tested mixtures reached the excellent class achieved by the designed renovation mortars. Similar studies were conducted by Cascione et al. [39], who examined clay plasters and lightweight gypsum, obtaining MBVs of 1.67 and 1.60 g/m^2^%RH, respectively. The obtained results are linked to macroporosity: ZSM-5, due to its porous structure, enhanced the porosity of the mortars, thereby improving their sorption capacity. The MBV results, combined with comparisons to other studies, demonstrate that the designed renovation plasters are a promising solution for passive regulation of indoor relative humidity. Higher total porosity and capillary absorption, together with excellent moisture buffering, can be explained by the intrinsic microporous structure of zeolites. Their fine and well-connected pores promote rapid adsorption–desorption cycles, enhancing the ability of the plaster to regulate indoor humidity despite the overall increase in porosity. In particular, mortars with 35% and 50% ZSM-5 replacement achieved MBVs above 2.1 g/m^2^%RH, clearly surpassing the reference sample (1.92 g/m^2^%RH) and confirming the beneficial role of high-silica zeolites in moisture buffering performance.

**Figure 8:**
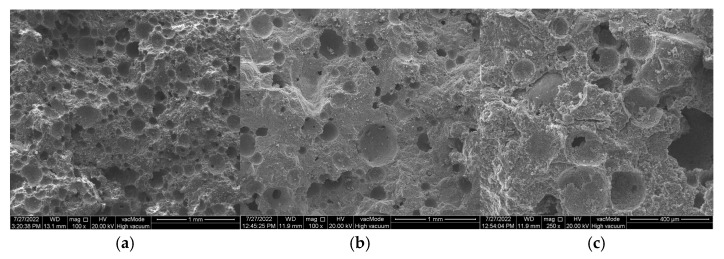
SEM images of renovation plasters with 35% zeolite replacement and the reference sample: (**a**) reference (R), (**b**) Na-A, (**c**) ZSM-5.

The MBV results also correlate with the water absorption coefficient data (Table 3). The reference mortar showed the lowest capillary rise (31 mm), while the plaster with 50% ZSM-5 absorbed 57% more water compared to the reference specimen. The incorporation of zeolite gradually increased water transport through the mortar structure and enhanced overall porosity. These findings suggest that the increase in porosity of the hardened mortars is caused by zeolite addition, and the observed microstructural differences arise from its intrinsic physical properties. Zeolites possess higher specific surface area and greater pore volume compared to cement. Differences are also evident between the zeolite types, which exhibit distinct framework structures and channel systems. The highest porosity was recorded for mortars containing ZSM-5, which exhibited the largest pore volume (0.1 cm^3^/g), as illustrated in Figure 8.

The total porosity of mortars is one of the most important parameters related to the control of water absorption by the material [40,41]. According to the WTA guidelines, the air-void content in fresh plaster mortar must exceed 25%, while the total porosity of the hardened plaster mortar must be greater than 40%. All designed renovation plaster formulations fulfilled these requirements. With increasing zeolite replacement, the total porosity increased. The highest total porosity was recorded for the samples with 50% ZSM-5 replacement (51.9%). The zeolite structure enhances the pore volume within the hardened matrix, enabling the crystallization of water and harmful salts within the plaster pores without damaging the plaster structure or the wall. This leads to improved frost and salt resistance. Similar properties were reported by researchers who used brick powder as a pozzolanic additive in cements [42,43].

### 3.2. Measurements of Electrical Resistivity

**Figure 9:**
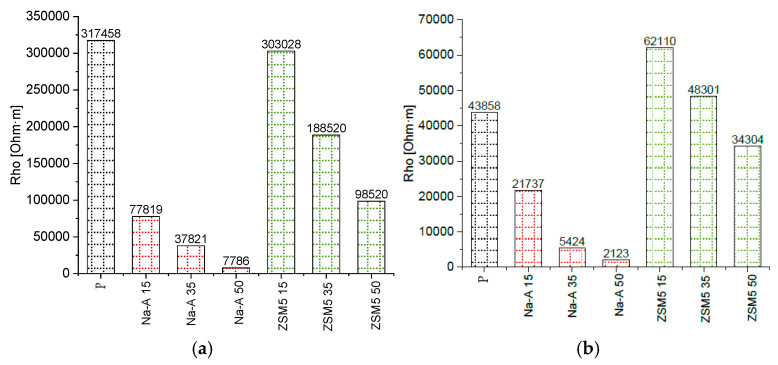
Resistivity values for various samples (**a**) at the frequency of 1 kHz, (**b**) at the frequency of 10 kHz.

Electrical resistivity measurements provided additional insights into the microstructure of the investigated mortars. The resistivity results of all specimens were compared at different frequencies. Bar charts of resistivity values for all specimens at 1 kHz and 10 kHz are presented in Figure 9. In all samples, a distinct decrease in resistivity was observed with increasing frequency. For example, between 1 kHz and 10 kHz, resistivity decreased approximately 7.2-fold for the reference mortar (317,458 → 43,858 Ω·m), 4.9-fold for ZSM-5 with 15% replacement (303,028 → 62,110 Ω·m), and 3.6-fold for Na-A with 15% replacement (77,819 → 21,737 Ω·m). Such a decrease in resistivity with increasing frequency is consistent with previous reports, which demonstrated that at higher frequencies the influence of electrode polarization diminishes, while frequency-dependent conduction mechanisms, such as Maxwell–Wagner interfacial polarization, become more pronounced [44].

The comparison of different mixtures showed that mortars containing the high-silica zeolite ZSM-5 exhibited significantly higher resistivity than their counterparts with the low-silica zeolite Na-A at the same replacement level. For example, at 1 kHz the mortar with 15% ZSM-5 reached 303,028 Ω·m, which is nearly four times higher than the value for Na-A 15% (77,819 Ω·m). At 10 kHz, this difference decreased but remained substantial (62,110 vs. 21,737 Ω·m). The reference mortar exhibited the highest resistivity at 1 kHz, whereas the mortar with 15% ZSM-5 showed the highest resistivity at 10 kHz. Within a given zeolite type, increasing the replacement level consistently reduced resistivity (e.g., ZSM-5 15% > ZSM-5 35% > ZSM-5 50%), suggesting enhanced connectivity of electrolyte-filled pores and intensified ionic conduction with higher zeolite content. The very low resistivity of mortars containing Na-A, particularly at 50% replacement, can be attributed to the high sodium content of this zeolite, which increases the conductivity of the pore solution and, together with the higher porosity, facilitates ion transport [45]. The obtained results are consistent with studies on alkali-activated systems and cement-based composites with zeolite additions. Zhang et al. [46] demonstrated that zeolite admixtures in alkali-activated slag binders reduce surface electrical resistivity due to intensified ionic conduction, whereas Girskas et al. [47] emphasized that the effect of zeolite strongly depends on the Si/Al ratio: low-silica zeolites decreased resistivity by enriching the pore solution with ions, while high-silica zeolites contributed to increased resistivity by densifying the matrix. These observations are in agreement with the present findings: mortars with Na-A exhibited lower resistivity than those with ZSM-5, although the difference diminished with increasing frequency.

**Figure 10:**
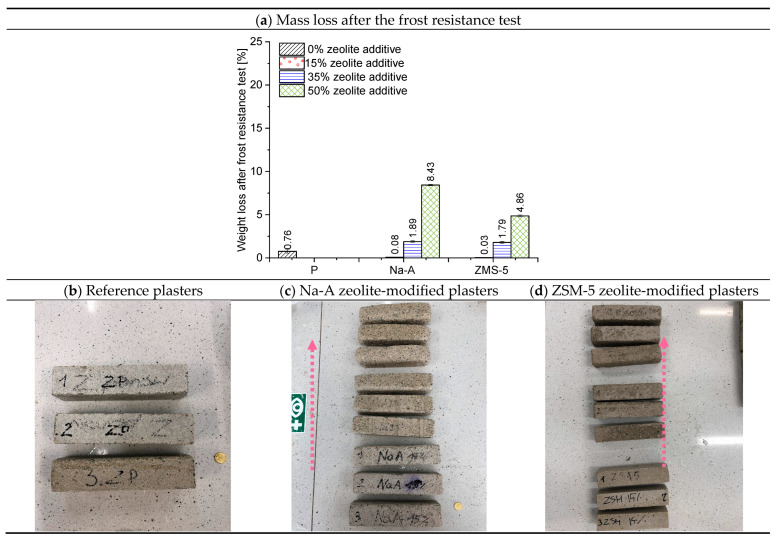
Average percentage mass loss of plaster specimens after frost resistance testing and the condition of their surfaces (arrow indicates increasing zeolite replacement: 15%, 35%, 50%).

**Figure 11:**
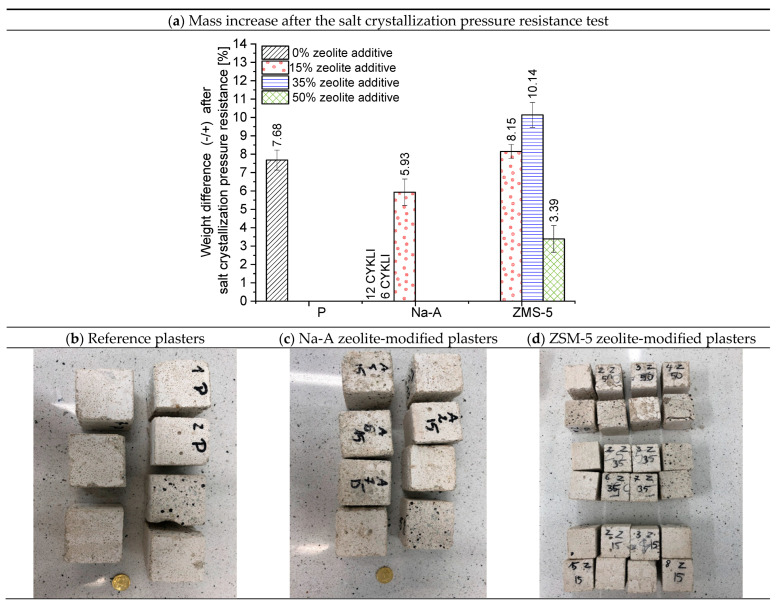
Average percentage mass increase in plaster specimens after the salt crystallization pressure resistance test and the condition of their surfaces.

A comparison of the resistivity of the mortars confirmed differences associated with microstructural modifications induced by the additives. Specimens with high-silica ZSM-5 showed somewhat higher resistivity than their counterparts with Na-A. At 1 kHz, the ZSM-5 15% mortar exhibited a resistivity approximately 20% higher than Na-A 15%. This difference decreased at higher frequencies, suggesting that the pore structure of ZSM-5 mortars is more compact, with fewer continuous electrolyte-filled pores. In addition, the zeolite replacement level was found to have a marked effect: the sample with 50% ZSM-5 displayed significantly lower resistivity than the 15% ZSM-5 mortar. This is likely due to the fact that at very high cement replacement, the cementitious matrix becomes less compact (with reduced C-S-H formation, more electrolyte-filled pores, and possibly unreacted zeolite grains facilitating ion migration), which decreases the electrical resistance. Overall, higher resistivity indicates a more compact and insulating microstructure. The results confirm that ZSM-5 zeolite additions promote the development of a favorable microstructure (better hydrated, rich in C-S-H, with fewer continuous portlandite-filled pores), whereas excessive replacement (50%) reduces structural compactness. Importantly, the observed increase in resistivity for mortars with high-silica ZSM-5 correlated with improved frost resistance and salt crystallization resistance (Figure 10 and Figure 11), confirming that this electrical parameter reflects both microstructural densification and enhanced durability. This underlines the potential of resistivity measurements as a reliable non-destructive quality assessment method for renovation plasters.

### 3.3. Mechanical Properties of the Renovation Plasters

**Figure 12:**
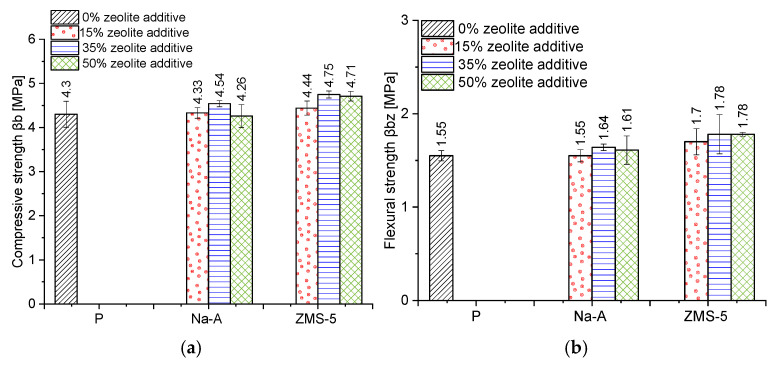
(**a**) Compressive strength [MPa], (**b**) Flexural strength [MPa].

The WTA Guideline 2-9-04 specifies the compressive strength range required for renovation plasters (1.5–5 MPa). Excessively strong renovation plasters may restrict the movements of the masonry layer and lead to harmful stresses, which can cause damage to the wall [48]. All formulations met these requirements (Figure 12). The highest compressive and flexural strength values were obtained for renovation plasters with ZSM-5 zeolite (compressive strength: ZSM-5 15%—4.44 MPa, ZSM-5 35%—4.75 MPa, ZSM-5 50%—4.71 MPa), with the optimum performance at 35% replacement. This result aligns well with the observation that the type and composition of zeolite (particularly the Si/Al ratio) strongly influence pozzolanic reactivity and the microstructure of the paste: high-Si/Al zeolites bind Ca(OH)_2_ more rapidly and effectively, promoting the formation of dense calcium silicate hydrate (C–S–H) and sealing of the interfacial transition zone (ITZ), especially after 28–90 days [49,50]. The ZSM-5 zeolite was characterized by the highest Si/Al ratio (90) and the highest content of reactive silica (92.05%) among the sodium zeolites used in the study, as well as the largest specific surface area among the applied pozzolanic additives (SBET = 369.1 m^2^/g). Ozen et al. [51] demonstrated that the specific surface area of zeolite affects pozzolanic reactivity mainly in the early reaction stage (<3 days), but has no significant effect on the reaction kinetics at longer curing times. Therefore, it can be inferred that in this case, the improvement in strength is primarily attributed to the high Si/Al ratio, as also reported by other researchers [52].

**Figure 13:**
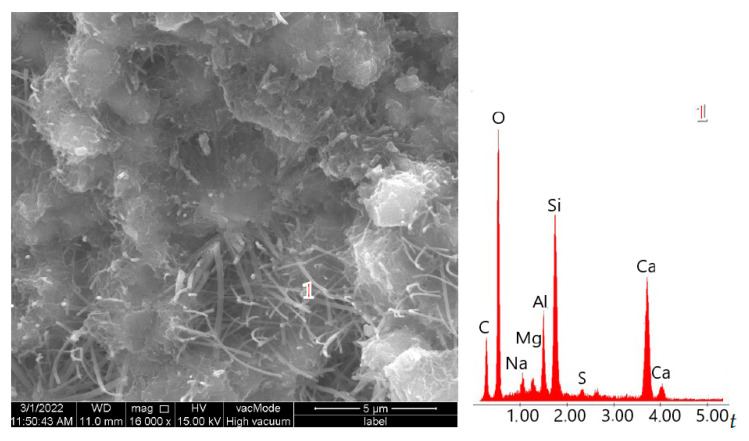
SEM micrographs of cement pastes with 35% ZSM-5 replacement.

**Figure 14:**
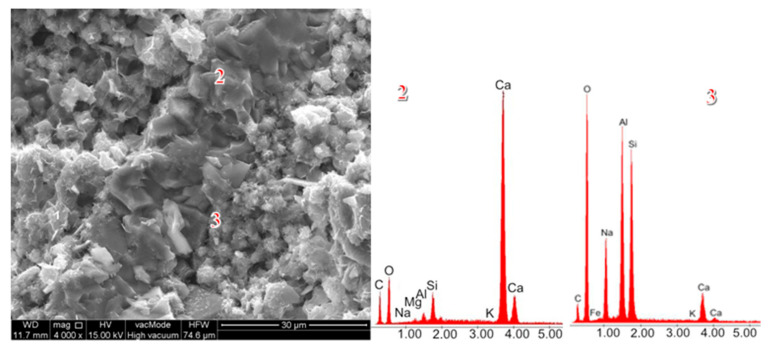
SEM micrographs of cement pastes with 50% ZSM-5 replacement.

Reactive SiO_2_ and Al_2_O_3_ react with Ca(OH)_2_ to form the calcium silicate hydrate (C–S–H) phase, which is responsible for high strength [53,54]. A higher amount of reactive silica and an increased Si/Al ratio resulted in a denser C–S–H phase with the desirable fibrous morphology. This was confirmed by SEM observations of the pastes (Figure 13 and Figure 14). In the samples with 35% ZSM-5 replacement (Figure 13), the structure appeared more compact, with large clusters of portlandite plates being difficult to identify. The microstructure was dominated by fibrous C–S–H (C–S–H I according to Diamond [55]), which contributes to the enhanced strength of the plasters.

Replacing 50% of cement with Na-A zeolite resulted in the formation of portlandite clusters (Figure 14, EDS point 2), which led to a reduction in strength. It is widely recognized in the literature that large portlandite crystals weaken the mechanical properties of mortars and concretes. The presence of Ca(OH)_2_ in the porous interfacial transition zone (ITZ) between paste and aggregate, as well as in large pores, contributes to the development of factors that reduce strength. All specimens with 50% zeolite replacement exhibited the lowest strength values. This is attributed to the excessive zeolite dosage, and consequently to the reduced amount of binder responsible for cohesive and strength-providing properties (dilution effect). The 50% zeolite replacement also caused the C–S–H phase to become highly enriched in aluminum, thereby diminishing its mechanical performance (Figure 14, point 3).

### 3.4. Frost Resistance

Frost resistance is one of the key factors determining the durability of external plasters against corrosion [56]. The percentage mass loss of the plasters after testing and the condition of the specimens are presented in Figure 10. With increasing zeolite replacement (the arrow in the photographs indicates the increasing amount of zeolite), the surface became progressively rougher. The specimens lost their angular shape—the edges became rounded and the aggregate became more visible. The analyzed renovation plasters without zeolite, as well as those with 15% and 35% zeolite replacement, demonstrated high frost resistance. In these cases, the mass loss did not exceed 2%. The best performance was achieved by plasters containing ZSM-5 zeolite. The mass loss values were as follows: ZSM-5 15%—0.03%, ZSM-5 35%—1.79%, and ZSM-5 50%—4.86%.

### 3.5. Salt Crystallization Resistance

The results of the salt crystallization resistance test are expressed as the percentage relative mass change after the test compared to the initial mass of the specimen, and as the number of cycles after which disintegration occurred, interpreted as the loss of resistance to salt crystallization pressure. The percentage mass increase in the plasters and the surface condition of the outer layer of the renovation plasters after the salt resistance test are presented in Figure 11. The best performance was achieved by mortars containing ZSM-5 zeolite. All specimens with this high-silica zeolite withstood 15 test cycles. This indicates good water absorption capacity and effective accumulation of crystallizing salts within the pores, combined with high strength associated with the formation of fibrous, silica-rich calcium silicate hydrate (C-S-H). The highest mass increase was recorded for specimens with 35% ZSM-5 replacement (10.14%), which corresponds to the greatest salt crystallization within the pores of these plasters. The specimens with 15% ZSM-5 replacement exhibited an 8.15% mass increase, whereas those with 50% replacement showed a mass increase of 3.39%. The surface condition of the specimens with 50% ZSM-5 (Figure 11d) revealed cracking and flaking. These specimens absorbed a considerable amount of salt; however, their strength was insufficient to withstand the crystallization pressure, leading to cracking. Consequently, the mass increase was not as high as in the 35% ZSM-5 specimens, since salt uptake was accompanied by mass loss due to progressive degradation. Similar to the results of compressive/flexural strength and frost resistance testing, the aluminum-rich C-S-H phase (Figure 11) lost its mechanical integrity. The poorest results were obtained for mortars containing Na-A zeolite. Only the specimens with 15% replacement survived until the end of the test. The 35% Na-A specimens failed after 12 cycles, while those with 50% replacement endured only 6 cycles.

Mass loss was not dependent on open porosity. Zeolite is a porous material that intensively absorbs water from the cement paste. The structure of the interfacial transition zone (ITZ) in these mortars will therefore differ from that in traditional mortars [57]. Smarzewski et al. [58] reported that mass loss of specimens under the influence of salts is not always directly related to open porosity. Instead, it is primarily associated with the strength of the ITZ itself, i.e., the quality of the bond between the paste and the aggregate.

### 3.6. Adhesion

**Figure 15:**
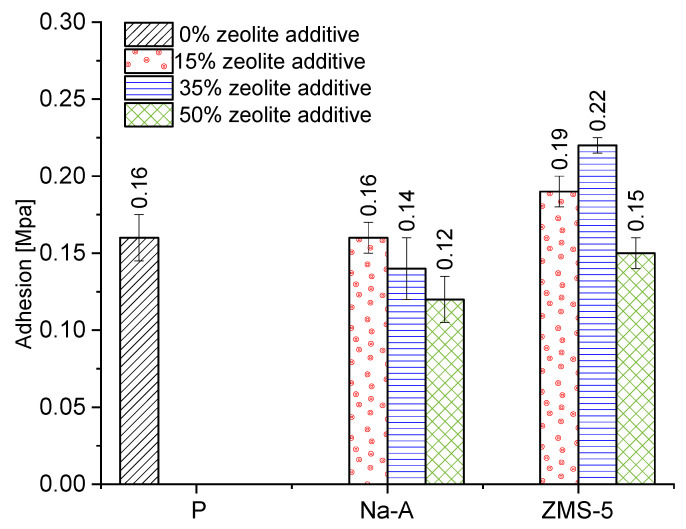
Adhesion.

Adhesion is defined as the force or work required to separate two contacting bodies. The discussed force is expressed per unit of contact area. Cohesion, in turn, refers to the force acting between molecules, generated and operating within a given substance. It consists of van der Waals forces (intermolecular attractive forces) and the mutual bonding of polymer molecules. Cohesive forces increase with the precision of the material’s fit and the tighter arrangement of molecules relative to each other [59,60]. These forces may cause significant damage to the brick substrate [4]. Therefore, the selection of mortar must be compatible with the properties of the substrate. Among the analyzed plasters, the highest adhesion was exhibited by those with ZSM-5 zeolite at 15% (0.19 MPa) and 35% (0.30 MPa) replacement levels (Figure 15). The poorest bond strength to the substrate was recorded for mortars with 50% zeolite replacement, with values ranging between 0.12 and 0.15 MPa. Similar trends were observed in the study by Barnat-Hunek and Klime [4], who investigated the adhesion of plasters with clinoptilolite to ceramic brick.

## 4. Conclusions

Synthetic zeolites Na-A and ZSM-5 showed clear differences in structural and physicochemical properties, which directly influenced plaster performance. Na-A (LTA) had a low Si/Al ratio of 0.85, pore volume of 0.03 cm^3^/g, pore diameter of 3.4 nm, and a specific surface area of 11.9 m^2^/g. In contrast, ZSM-5 (MFI) was characterized by a high Si/Al ratio of 90.0, a pore volume of 0.10 cm^3^/g, a pore diameter of 3.2 nm, and a specific surface area of 369.1 m^2^/g. These values explain the higher reactivity of ZSM-5, which provided stronger pozzolanic activity than Na-A.

The addition of zeolites modified the pore structure of the mortars. The reference plaster had an apparent density of 1307 kg/m^3^ and porosity of 44.0%. With 15% zeolite addition, density slightly increased (Na-A 1332 kg/m^3^, ZSM-5 1321 kg/m^3^), but at higher replacement levels, it decreased while porosity grew. The highest porosity (51.9%) was recorded for ZSM-5 50%. This more open structure translated into higher capillary absorption: the reference absorbed 7.4 kg/m^2^ (31 mm rise), while Na-A 50% absorbed 13.4 kg/m^2^ (45 mm rise) and ZSM-5 50% absorbed 14.4 kg/m^2^ (70 mm rise). These results demonstrate that zeolite-modified plasters can nearly double their capillary absorption capacity, a beneficial effect in renovation plasters, where controlled moisture removal and salt accumulation are desired.

Zeolite additions also improved hygroscopic behavior. The Moisture Buffer Value (MBV) increased from 1.92 g/m^2^%RH in the reference mortar to 2.1 g/m^2^%RH with Na-A, and above 2.3 g/m^2^%RH for ZSM-5 at 35–50% replacement. All modified mortars reached the “excellent” MBV class, confirming their potential for passive indoor humidity regulation and microclimate improvement.

Mechanical and durability performance was enhanced by ZSM-5. Compressive strength reached 4.44 MPa at 15% replacement, 4.75 MPa at 35%, and 4.71 MPa at 50%, compared to the WTA guideline range of 1.5–5.0 MPa. Frost resistance was highest for ZSM-5 plasters, with mass losses of 0.03% (15%) and 1.79% (35%), versus nearly 2% for the reference. Salt crystallization resistance was also best for ZSM-5, with 35% replacement specimens surviving 15 cycles and showing a mass increase of 10.14%.

The microstructural role of the Si/Al ratio was decisive. SEM observations of mortars with 35% ZSM-5 replacement revealed dense fibrous C-S-H phases with limited portlandite, corresponding to higher strengths. In contrast, Na-A mortars showed more C-A-S-H phases and portlandite clusters, which weakened their structure. This demonstrates that high-Si/Al zeolites, like ZSM-5, are more effective in densifying the matrix, while low-Si/Al zeolites, such as Na-A, promote less favorable hydration products. Resistivity measurements confirmed the significant influence of zeolite type and dosage on mortar morphology of cement pastes. Mortars with high-silica ZSM-5 exhibited higher resistivity than those with low-silica Na-A, reflecting their denser and more insulating microstructure. Na-A mortars showed very low resistivity, particularly at 50% replacement, which can be attributed to the high sodium content enhancing pore solution ionic conductivity, along with increased porosity, facilitating ion transport. At the same time, higher replacement levels of both zeolites reduced resistivity, indicating weakened matrix integrity at excessive cement substitution. The results demonstrate that resistivity measurements effectively reflect microstructural differences and can serve as a useful, non-destructive tool for assessing the durability of renovation plasters.

Although synthetic zeolites may increase material cost, their improved durability, resistance to salt crystallization, and excellent moisture buffering capacity suggest that their use could be cost-effective in conservation practice, where long-term performance and reduced maintenance are priorities. From a practical perspective, the investigated mortars are particularly suited for damp historic masonry in temperate and humid climates, where resistance to moisture and salts is crucial. Future research will focus on extending the approach to natural zeolites, alternative binders, and in situ exposure studies under real environmental conditions.

## Figures and Tables

**Table 1:** Mix compositions of the renovation plasters.

	P	Na-A 15%	Na-A 35%	Na-A 50%	ZSM-5 15%	ZSM-5 35%	ZSM-5 50%
CEM I 52.5R	15.5	13.2	10.1	7.75	13.2	10.1	7.75
Synthetic zeolite	-	2.3	5.4	7.75	2.3	5.4	7.75
Expanded clay	3.5	3.5	3.5	3.5	3.5	3.5	3.5
Hydrated lime	5	5	5	5	5	5	5
Quartz sand 0–2 mm	60.35	60.35	60.35	60.35	60.35	60.35	60.35
Zeolite clinoptilolite	15	15	15	15	15	15	15
Methylcellulose	0.15	0.15	0.15	0.15	0.15	0.15	0.15
Ethylene vinyl acetate copolymer	0.5	0.5	0.5	0.5	0.5	0.5	0.5

**Table 2:** Properties of zeolites [22].

	Na-A (LTA) Three-dimensional channel system: 4.1 × 4.1 Å Si/Al ratio = 0.85 Pore volume—0.03 cm^3^/g Pore diameter—3.4 nm Specific surface area SBET = 11.9 [m^2^/g]	ZSM-5 (MFI) Three-dimensional channel system: 5.1 × 5.6 Å Si/Al = 90.00 Pore volume—0.1 cm^3^/g Pore diameter—3.2 nm Specific surface area SBET = 369.1 [m^2^/g]
(a)	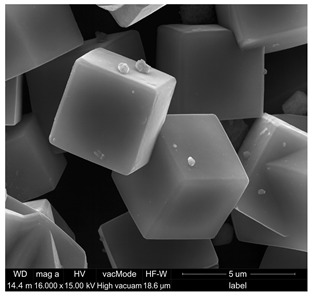	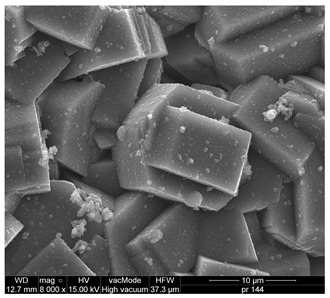
(b)	The synthesis was carried out using 0.5 dm^3^ of post-synthesis waste solution (ROZ), 1 dm^3^ of 1 M NaOH, and 10 g of aluminum foil, under the reaction temperature of 80 °C for 24 h.	The synthesis was carried out using 0.1 dm^3^ of post-synthesis waste solution (ROZ), 0.1 dm^3^ of a 3.5% aqueous solution of tetrapropylammonium bromide (TPABr), and 0.008 dm^3^ of 5 M sulfuric acid (VI), under the reaction temperature of 195 °C for 65 h.
(c)	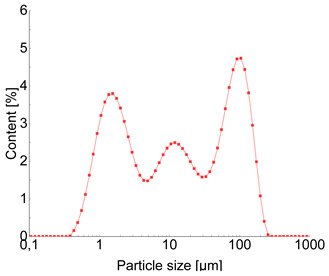	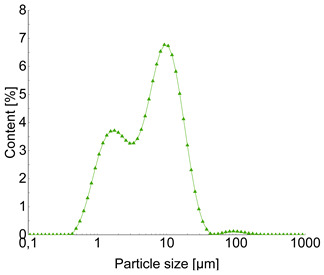

**Table 3:** Physical properties of the renovation plasters.

Physical Characteristics	P	Na-A 15%	Na-A 35%	Na-A 50%	ZSM-5 15%	ZSM-5 35%	ZSM-5 50%
Distribution [mm]	175	172	169	165	170	168	165
Density of fresh mortar [kg/m^3^]	1435	1501	1508	1532	1510	1513	1542
Apparent density ρ_a_ [kg/m^3^]	1307	1332	1311	1282	1321	1294	1280
Specific density ρ [kg/m^3^]	2390	2380	2360	2328	2420	2430	2470
Tightness [%]	0.54	0.56	0.55	0.55	0.54	0.53	0.51
Total porosity [%]	44.0	45.0	45.4	46.4	45.4	47.7	51.9
Contents of pores in fresh air render [%]	28.1	27.4	26.8	25.1	27	26.2	26.3
Factor water absorption [mm]	31	32	36	45	34	40	70
Factor water absorption *C_m_* [kg/m^2^]	7.4	7.1	9.5	13.4	8.2	11.4	14.4

## Data Availability

The original contributions presented in this study are included in the article. Further inquiries can be directed to the corresponding author.
